# Prevalence and Predictors of Depression in People living with HIV/AIDS Attending an Outpatient Clinic in Nigeria

**Published:** 2014

**Authors:** Adetunji Obadeji, Adegboyega O. Ogunlesi, Timothy O. Adebowale

**Affiliations:** 1 Department of Clinical Services, Neuropsychiatric Hospital, Aro, Abeokuta, Nigeria.; 2 Department of Clinical Services, Neuropsychiatric Hospital, Aro, Abeokuta, Nigeria.

**Keywords:** Depression, HIV/AIDS, Nigeria, Prevalence

## Abstract

**Objective:** Studies have identified high prevalence of depression among people living with HIV/AIDS, but only few studies have looked into this association in this environment. The objectives were to determine the prevalence of major depressive disorder, associated socio-demographic and psychosocial variables in people living with HIV/AIDS attending an out-patient clinic at the Olabisi Onabanjo Teaching Hospital (OOUTH), Sagamu, Nigeria.

**Methods:** A cross-sectional survey was carried out on one-hundred and thirty subjects living with HIV/AIDS attending out-patient clinic at the OOUTH, Sagamu. They were assessed with a socio-demographic questionnaire designed by the researchers, and they also had a clinical interview with the depression module of the Structured Clinical Interview Schedule for Axis 1 DSM-IV disorders (SCID). The diagnosis was made according to the DSM- IV criteria and severity assessed with the Hamilton Rating Scale for depression.

**Results:** The prevalence of depression was 23.1% and was higher than figures reported in the general population studies in Nigeria. Of this proportion 46.7%, 50%, 3.3% were mildly, moderately and severely depressed. Majority (40%) were within the 30-39 years range. Women accounted for 69.2% of the study population and 46.9% of the subjects were either divorced/separated or widowed. Depression was significantly associated with being female and having suicidal thoughts or attempt. There was no association between marital status, disclosure of status and duration of HIV diagnosis.

**Conclusion:** The prevalence of depression is high among people living with HIV/AIDS in Nigeria. An appropriate mental health intervention programme would be necessary as part of national programme for people living with HIV/AIDS to reduce the negative impact of depression on them.

**Declaration of interest:** None.

## Introduction

There is increasing evidence that major depression impacts negatively on the course of HIV infection, yet few studies have looked at the prevalence of depression and its associated socio-demographic predictors in people living with HIV in this environment. Depression has been reported to be the most common mental illness among people living with HIV/AIDS with the prevalence ranging from 0-47.8% (1, 2). These variations have been attributed to a number of factors, including differential assessment strategies, varying recruitment approaches and other methodological issues. Studies from Western countries have shown that the prevalence of depression is higher in people living HIV than in HIV-negative controls. Several factors such as social stigma, occupational disability, isolation from social support, long-term physical discomfort and illness have been attributed to this increase (3-5). This state of chronic stress for infected individuals leaves the person with psychological adjustment to living with incurable disease. 

Depression, however, continues to be the most observable psychiatric disorder among people living with HIV/AIDS and other chronic and incurable diseases (6, 7). It imparts negatively on the disease (8-10). Besides, it is associated with HIV risk behaviour and influence adherence to highly active antiretroviral therapy (HAART) (11-13). Evidence suggests that psychosocial factors such as stress and depression may have harmful impact on the course of many diseases such as cancer and cardiovascular disease (14). Studies examining the effect of depression on HIV disease have demonstrated association between depression and early, as well as late disease progression in men and between depression and mortality in women (8). Alteration in cellular immunity has been shown in depressed individuals who are otherwise healthy (15). Thus, the mechanisms by which stress and depression may influence disease progression and mortality in HIV infection remain to be determined (2).

Besides mental illness complicating HIV infection, studies have shown that the prevalence of HIV among mentally ill subjects is seven times higher than in the general population (16). People living with mental illness are particularly vulnerable to infection with HIV because of several factors including higher prevalence of poverty, homelessness, high risk sexual activities and abuse, and social isolation (12, 16, 17). The diagnosis of depression also may be complicated by the fact that both depression and HIV infection result in similar somatic or physical symptoms. Fatigue, loss of weight, low appetite, and reduced libido may be either manifestation of HIV-related illness or depression. However, cognitive and affective symptoms such as sad feeling, loss of interest in formerly enjoyable activities, guilt, and irritability are components of mood alone (6). Distinction has to be made between physical symptoms arising from HIV infection progression and those due to depression.

The outcome of HIV among mentally ill subjects has been observed to be poorer (2, 8, 18). This has been attributed to several factors: poor adherence to HAART, lower accessibility to HAART and immunological changes associated with mental illness itself. 

Nigeria remains one of the most affected geographical areas by the epidemics. The resources are limited. However, there is dearth of information on the impact of mental illness particularly depression on the course and outcome of HIV/AIDS. Hence, there need to determine the prevalence of depression and its predictors among people living with HIV/AIDS in this environment. 

## Materials and Methods

A cross-sectional study was carried out on the subjects attending the HIV out-patient Clinic of the Olabisi Onabanjo University Teaching Hospital, Sagamu, Nigeria in Jan 2009-June 2009. The target population was the entire HIV/AIDS patients who presented to the clinic for regular follow-ups. Each consecutive patient was recruited for the study until the sample size was gotten. Patients below the age of 18 years were excluded from the study, likewise those with debilitating illness such as head injury, medical illness unrelated to HIV(e.g., malignancy, renal failure, or chronic hepatitis), severe HIV- related conditions (i.e., current opportunistic infection), severe psychiatric illness and inability to function independently were excluded as well.

A questionnaire was developed to assess the socio-demographic and psychosocial variables. Presence of depression was assessed with the SCID for axis one Diagnosis. The diagnosis of depression was made with the fourth edition of the Diagnostic Statistical Manual (DSM-IV) by American Psychiatric Association (APA, 1994). The SCID has been used extensively in Nigeria (19, 20). The instrument has been shown to have a good reliability for categorical construct for DSM-IV diagnoses and good standard validity (21). The screening and diagnostic module on depression was used in this study. The severity of the depression was quantified using the 17-item Hamilton Depression Rating Scale (HDRS) among subjects who met the DSM-IV criteria for depression. The HDRS has demonstrated a bifactorial structure with factors corresponding to the HDRS subscales, an excellent internal consistency and test-retest reliability, a very good convergent validity, and acceptable discriminant validity. Strikingly, in contrast to the BDI, HDRS scores were found to be unconfounded by the presence of HIV symptomatology (22). All the questionnaires were administered by trainee psychiatrists who had earlier been trained in the administration of the instruments. Approval for research protocol was obtained from the Ethics and Research Committee of the Olabisi Onabanjo University Teaching Hospital, Sagamu. Informed consent was obtained from all the subjects after the objectives o f the study have been explained to them. 

Data were analysed using a statistical package for social sciences (SPSS) computer software version 11 for Windows (SPSS, Chicago, IL, USA). Appropriate data presentations were made, including frequency distribution, cross tabulations and statistical analysis with test of significant were carried out. The chi-square and t-test were used to test for association between depression and other parameters as deemed appropriate. A P value of less or equal to 0.05 was considered to indicate statistical significance.

## Results

One hundred and thirty subjects were interviewed for the study. Of the 130 interviewed, 30 (23.1%) met criteria for major depressive disorder, and of these, 46.7%, 50%, 3.3% were mildly, moderately, and severely depressed respectively. Majority (69.2%) of the subjects were female. The age of the respondents ranged between 19 and 64 years with a mean age of 37.74 years (SD = ±9.0). Fifty (38.5%) were married, 14.6% were never married, 14.6% were either divorced or separated and 32.3% were widows/widowers. Only 4.6% of the respondents had no formal education.

Table 1 shows the relationship between depression and socio-demographic variables. There was no statistical significant difference in the prevalence of depression among those who aged 40 years and younger and those who aged more than 40 years (p > 0.050). The difference in the mean age of those that were depressed and non-depressed was not significant (t = -0.920, p = 0.920). Major depression was found to be twice as common in the females compared with the male with a p-value of 0.043.

As shown in table 2, of forty-eight (36.9%) that admitted having repeated thoughts of suicide, 17 (34.4%) were depressed as against 13 (15.9%) of those without such thoughts. The association between depression and thought of suicide was significant at p = 0.011. Depression was also significantly associated with suicidal attempt (p = 0.001).

**Table 1:** Association between socio-demographic variables and frequency of depression

**Variable**	**n**	**Depression n(%)**		**P value**
		**Depressed**	**Not depressed**	
**Age**				
< 40 years	88	21(23.9)	67(76.1)	0.472
> 40 years	42	9(21.4)	33(78.6)	
**Mean age**	37.75	39.20	37.31	0.085
**Sex**				
Male	40	5(12.5)	35(87.5)	0.043
Female	90	25(27.8)	65(72.2)	
**Marital status**				
Never married	19	4(21.1)	15(78.9)	0.386
Widowed	42	13(31.0)	29(69.0)	
Separated/divorced	19	5(26.3)	14(73.7)	
Married	50	8(16.0)	42(84.0)	
**Religion**				
Christianity	97	26(26.8)	71(73.2)	0.084
Islam	33	4(12.1)	29(87.9)	
**Educational level**				
No formal education	6	2(33.3)	4(66.7)	0.905
Primary school	37	9(24.3)	28(75.7)	
Secondary and higher	87	9(22.9)	68(77.1)	
**Number of people living with**				
None	9	4(44.4)	5(55.6)	0.288
≤ 4	70	15(21.4)	55(78.6)	
> 4	51	11(21.6)	40(78.4)	

**Table 2:** Depression and psychosocial variables

**Variable**	**Total (N=13)**	**Depression n(%)**		**P value**
		**Depressed**	**Not depressed**	
**Disclosure of status**				
Yes	110	23(21.9%	87 (79.1%)	0.169
No	20	7(35.0%)	13(65.0%)	
**Get expected support**				
Yes	87	20(23.0%)	67 (77.0%)	0.297
No	23	3(13.0%)	20(87.0%)	
**Thought of ending it all**				
Yes	48	17(35.4%)	31(64.6%)	0.011
No	82	13(35.4%)	69(84.1%)	
**Suicide attempt**				
Yes	9	6(66.7%)	3(33.3%)	0.001
No	121	24(19.8%)	97(80.2%)	
**Sex with stranger**				
Yes	19	3(15.8%)	16(84.2%)	0.415
No	111	27(24.3%)	84(75.7%)	
**Sex with commercial sex worker**				
Yes	11	2(18.5%)	9(81.8%)	0.687
No	119	28(23.5%)	91(76.5%)	
* **Number of sexual Partner ever**				
One	44	9(20.5%)	35(79.5%)	0.415
More than one	84	21(25.0%)	63(75.0%)	
**Guilt of previous Sexual practices**				
Yes	28	8(28.6%)	20(71.4%)	0.436
No	102	22(21.6%)	80(78.4%)	
**Duration of diagnosis**				
≤ 1 year	39	8(20.5%)	31(79.4%)	0.650
> 1 year	91	22(24.2%)	69(75.8%)	

* Those with no sexual partner ever were not included in the analysis

## Discussion

This study looked at the prevalence of depression and the associated psychosocial features of people living with HIV/AIDS among one-hundred and thirty subjects. In this study, about a quarter of people living with HIV/AIDS were depressed. This finding is similar to that of Morison et al. in North Central Florida and Bolton et al. in Southwest Uganda in which the authors reported rates of 19.4 and 21%, respectively (2, 23). However, our finding is relatively lower than the 36.2% and 38% reported by Turrina et al. in Brescia, Italy and Lawler et al. in Botswana, respectively (24, 25). Rates ranging from 0%-47.8% had been reported in the literature (1). These variations have been attributed to several factors. These include (i) methodological differences with respect to subject selection, intervention measures, and instruments used; and (ii) patient characteristics such as sexual orientation, race, and intravenous drug use. For example, the study by Turrina et al. was solely among intravenous drug users. The stage of the disease may also be a factor. However, the study by Morrison et al. did not find any significant difference between HIV seropositive and seronegative individuals (2). However, when compared with the general population, the prevalence reported in this study is quite high. For example, in a life time and 12-month prevalence study by Gureje et al., the 12-month and life time prevalence for major depressive disorder was 1.0 and 3.3%, respectively (26). This showed that depression was about 23 times more common in people living with HIV/AIDS compared with the general population. The prevalence of depression however, was similar to that obtained with people with other chronic medical such as diabetes or cancer (20). 

There are several possibilities for this high fold increase. Firstly, with the introduction of HAART, HIV infection becomes a chronic disease with associated distress of living with a chronic illness. Secondly, patients with HIV/AIDS possibly suffer more losses, in form of death of spouse, children, and even loss of friends and relatives support as a result of stigma. It is also possible that, most anti-retroviral medications may play a role in the etiology of depression (i.e., having clinical depression as one of their side effects) (27). Distress from the physical complications of the disease may also play a role. Prevalence of depression had been shown to be high among medically ill patients (28). The possibility of over diagnosis due to similarity between depressive symptoms and physical symptoms of HIV is another factor (7). 

With the exception of gender, our study did not find any association between major depression and socio-demographic variables. Depression was found to be twice common in female compared with the males. This is similar to what obtained in the general population without physical illness (29, 30). Many of the subjects in this study were found to be living without their spouses, either as separated, widowed or divorced. Nearly half of the subjects were widowed. The high proportion of widowhood is worrisome as this reflects adverse effect on family. In contrast to what obtained in the general population, no association was found between depression and marital status.

Majority of the patients in this study had disclosed their HIV status similar observation by Arnold et al. (31). And of this group, most were satisfy with the support they were getting from others. However, absence of expected support did not predict development of depression as reported by Schrimshaw (32). About half of the respondents reported repeated suicidal ideas and a significant number (6.9%) had attempted suicide similar to 8.7% reported by Olley among HIV/AIIDS population in South Africa (33). In this study a strong association was found between suicidal ideas, and suicidal attempt and major depression. Similar findings have been reported by other authors (33, 34). This possibly, may be due to the devastating effect of the disease and the fact that HIV/AIDS remains incurable. It may also be due to the fact that suicidal ideas and attempt are features of major depression.

In conclusion, the prevalence of major depression is high among people with HIV/AIDS in this part of the world. Health care providers should maintain high index of suspicion regarding the possibility of depression among HIV/AIDS patients. Also, there is need to incorporate mental health services into the national HIV programmes to reduce the negative impact of depression on people living with HIV/AIDS in Nigeria. 

***Limitation***

This is a cross-sectional study looking at the prevalence of depression among people living with HIIV/AIDS. The fact that this study did not have controls makes it difficult to determine causality.

## Authors’ contributions

AO conceived and designed the evaluation and drafted the manuscript. He was also involved in collection and interpretation of clinical data and in revision of the manuscript. AOO participated in designing the evaluation, data analysis and in the revision of the manuscripts. TOA re-evaluated the manuscript, re-analysed the clinical and statistical data and revised the manuscript. All authors read and approved the final manuscript. 
